# The Attitude of the General Dentist in the Republic of Croatia toward Treating Children

**DOI:** 10.3390/children9121888

**Published:** 2022-12-01

**Authors:** Lidia Gavić, Ivana Nikolić, Sharanbir K. Sidhu, Daniel Jerković, Antonija Tadin

**Affiliations:** 1 Study of Dental Medicine, School of Medicine, University of Split, 21000 Split, Croatia; 2 Study of Dental Medicine, Department of Maxillofacial and Oral Surgery, University Hospital of Split, 21000 Split, Croatia; 3 Barts & The London School of Medicine and Dentistry, Institute of Dentistry, Centre for Oral Bioengineering London, Queen Mary University of London, London E1 2AD, UK

**Keywords:** child dental treatment, dental care, dentist attitude, pediatric dentistry

## Abstract

The aim of this study was to determine the attitude of general dentists in the Republic of Croatia toward working with children. The questionnaire survey involved 361 general dentists. The questionnaire was divided into three parts and contained 34 questions. The first part of the questionnaire survey contained demographic data questions. In the second part, dentists answered questions about the characteristics of the dental offices where they work, the materials that they mostly use, and how they work with children. The third part of the questionnaire referred to attitudes about working and treating young children and consisted of 12 statements, to which the answers were offered on a Likert scale from one to five. This study showed that only 12.46% of dentists have a positive attitude toward treating children and 30.19% of dentists have a negative attitude toward treating children. In addition, the attitude toward working with children correlates with both the knowledge they acquired during dental studies (R = 0.355; *p* ≤ 0.001) and gender (R = −0.103; *p* = 0.035). This study confirmed that women have a more often positive attitude toward treating young children. There was a major correlation between the level of education and positive attitudes toward treating children. The child’s non-cooperative behavior was the main reason why general dentists refuse to work with children.

## 1. Introduction

Pediatric dentistry is a specific branch of dental medicine that requires knowledge and understanding of the physical and mental development of the child [1]. The main task of pediatric dentistry is to promote and care for the health of the stomatognathic system in children, with the relevant education and motivation of parents about the importance and opportunities to preserve oral health [1,2]. Pediatric dentistry provides primary and comprehensive oral health prevention and treatment in infants and children, including adolescents up to 18 years of age. This specialist branch requires skill and discipline and is specific because of the age of the patients. In addition, it requires understanding and knowledge of the characteristics of individual developmental stages of a child’s psychological development. The specialization of pediatric dentistry also includes the therapy of children with developmental and other disabilities [2]. Pediatric dentistry includes procedures and preventive measures to maintain optimal oral health [3].

The development of pediatric dentistry in the Republic of Croatia as a distinct, independent dental discipline began with the establishment of the Department of Pediatric and Preventive Dentistry at the Medical Faculty in Zagreb in September 1961, which is considered the beginning of this dental branch in Croatia [4].

One of the principles of working with children is the ability to positively guide the child during the dental procedure and create a comfortable feeling to improve the quality of oral health and achieve a positive relationship with the dentist. Therefore, it is crucial that all dentists, not only pediatric dentists, assess and recognize the psychological characteristics and reactions of the child, to determine the need to shape behavior and thus prevent dental fear and anxiety [5,6,7].

The European Academy of Pediatric Dentistry (EAPD) states that the main goals of undergraduate education are to create a dentist who will be capable and confident in most areas of pediatric dentistry and distinguish normal from pathological growth patterns. In addition, the purpose of this education is to train a dentist who will be part of a multidisciplinary team for child welfare. Future dentists must know about the child’s normal physical and mental development. They should be able to distinguish between biological and dental age, stages of sexual development, and the etiology and pathology of the most common diseases in children and adolescents. Furthermore, students are educated about the development of the craniofacial complex, skeletal deformities, and malocclusions. Students are expected to recognize and distinguish deciduous teeth from permanent ones and recognize various anomalies affecting permanent or deciduous dentitions [8,9].

This research aimed to determine the attitude of general dentists in the Republic of Croatia toward working with children. Furthermore, this study examined factors such as gender, age, knowledge, and experience that could potentially affect the attitudes of dentists toward working with children.

## 2. Materials and Methods

This research was conducted from November 2020 to April 2021 through an online questionnaire (Google form) distributed to dentists in the Republic of Croatia. The research was approved by the Ethics Committee of the Medical Faculty of the University of Split (Class: 003-08/21-03/0003, Reg.no.:2181-198-03-04-21-0066). The research had no financial support.

The target population of the conducted research was general dentists in the Republic of Croatia employed in private surgeries or health centers. In the Republic of Croatia, a total of 5066 dentists are registered. With a 5% error limit, a 90% confidence interval, and a 50% response distribution, the minimum required sample size was 358.

The questionnaire survey was completely anonymous, and all respondents were informed in detail about the purpose of the questionnaire. It consisted of a total of 34 questions divided into three parts.

The first part of the questionnaire contained questions on general and demographic data of respondents (age, gender, year of graduation, and place of residence).

The second part of the questionnaire contained questions about the characteristics of the practice in which the respondents work, the way they work, and the therapeutic procedures they carry out on children, as well as the materials they use most often.

The third part of the questionnaire referred to attitudes about working with children. It consisted of a total of 12 statements to which the answers were offered in the form of a Likert scale from 1 to 5 (1: strongly disagree, 2: disagree, 3: partially agree, 4: mostly agree, and 5: totally agree). The questionnaire was adapted from the research of Garg et al. [10]. The coefficient of internal consistency (Cronbach’s alpha) was 0.722, which indicated very good reliability of the questionnaire [11].

### Statistical Analysis

Statistical analysis was performed by using SPSS software package (IBM Corp., Armonk, NY, USA). By descriptive statistical analysis, the basic statistical parameters (mean, standard deviation, minimum, maximum, median, and interquartile range) were determined. The method of basic statistics was used for a description of the population and the calculation of Spearman’s correlation coefficient. General regression model (GRM) was used as the assessment of the influence of the predictor variables (age, gender, age of service, employment, and education) on the dependent variable (attitudes to working with children). The results of GRM are expressed in the form of Pareto charts. The level of significance was set at 0.05 for all tests.

## 3. Results

Three hundred sixty-one dentists from the Republic of Croatia participated in this study. The age of the respondents ranged from 24 to 65 years, while the average age was 38.47 ± 10.53 years. The year of graduation ranged from 1979 to 2020. Respondents had between 0 to 40 years of service, an average of 12.66 ± 10.14 years. Regarding the gender distribution, 74.51% of the participants were women.

The largest percentage of respondents were employed in private practice as the owners of the practices (42.38%). On the other hand, the number of dentists working in health centers of health and private practices were almost the same, with (28.53%) in health centers and (29.08%) as part of the team in private practices. A total of 264 respondents (73.13%) had a contract with the Croatian Health Insurance Fund.

**Figure 1:**
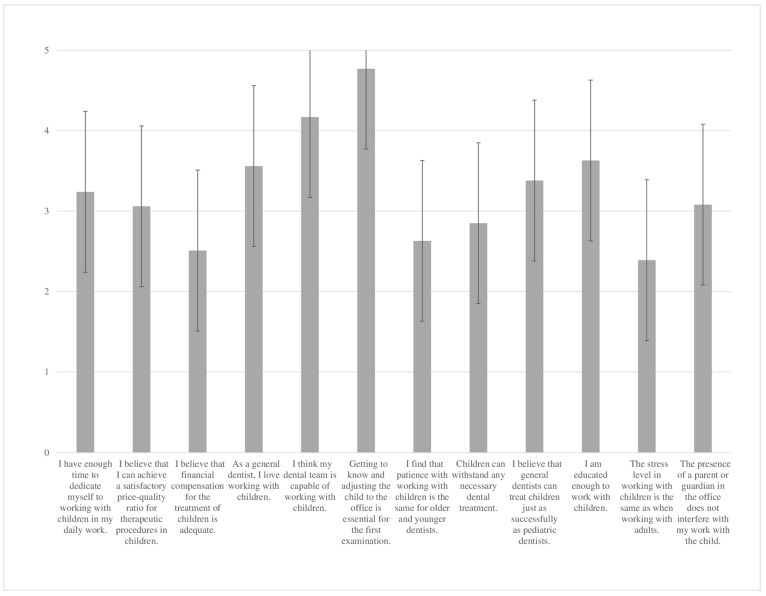
The distribution of answers on the attitude of dentists toward working with children.

The dentists’ scores on the questionnaire on their attitude to working with children ranged from 19 to 60, with a mean value of 39.02 ± 7.58. The distribution of answers on dentists’ attitudes toward working with children is shown in Figure 1.

According to Bloom’s classification, 12.46% of dentists have a positive attitude, i.e., they are willing to work with children, 57.34% of dentists were neutral, and 30.19% of dentists had a negative attitude, i.e., are less willing to work with children [12].

**Figure 2:**
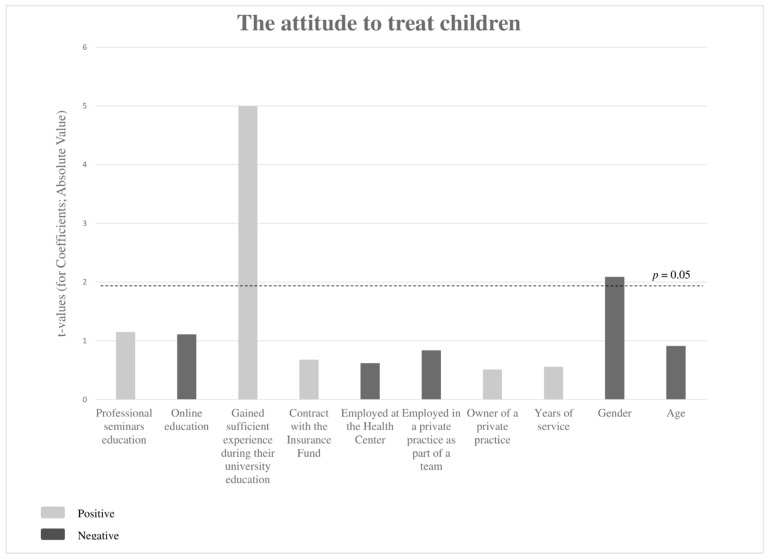
The dependence of the attitude to treat children based on prediction variables. The results of the linear regression analysis.

The results of the linear regression analysis are shown in Figure 2. Positive attitudes toward working with children depend on the gender of the respondents (β = −3.454, *p* = 0.039) and whether they gained sufficient experience in working with children during their university education (β = −6.809, *p* < 0.001).

The responses to questions on carrying out therapeutic procedures on children are presented in Table 1.

Of the preventive measures, the dentists surveyed most often performed fissure sealing (61.22%). In addition, fluoridation was performed by 130 dentists (36.01%). On the other hand, nutrition analysis was performed by only nine clinicians (2.49%), while only one (0.28%) stated that oral microflora in children was analyzed. A total of 252 (69.81%) dentists stated that the therapy of young permanent teeth is the most demanding when working with children. On the other hand, the treatment of deciduous teeth is considered the most demanding by 94 dentists (26.04%) and permanent teeth by only 15 practitioners (4.15%). In addition, when asked whether they perform endodontic treatment of deciduous teeth, 133 respondents (36.84%) answered positively, while 228 (63.16%) responded negatively. The “tell-show-do” is the most popular child behavior control technique used by 284 (81.44%) dentists. The distraction method was used by 30 dentists (8.31%), the voice control method by 23 (6.37%), the behavior support method by 22 dentists (6.09%), and four dentists stated that they used something else without specifying what.

The majority of the respondents (57.10%) felt that they did not gain enough experience working with children during their university education. When asked whether their experience of working with children improved after completion of their university education, 42.37% of respondents answered affirmatively: via the Internet (6.84%), through professional seminars (8.10%), by working in practice (17.7%), and in some other way (6.64%).

### Spearman’s Correlation Analysis

According to the gender variable, women are more often employed in health centers (R = −0.124; *p* = 0.026), and men more often have the opinion of gaining enough experience working with children during university (R = 0.138; *p* = 0.013). The attitude toward improving education by working in practice negatively correlates with education in professional seminars (R = −0.440; *p* < 0.001) and searching for the additional literature on the Internet (R = −0.383; *p* < 0.001). The age of the respondents is positively correlated with education about working with children at professional seminars (R = 0.288; *p* = 0.012), and using voice control in working with children (R = 0.124; *p* = 0.026), and negatively correlated with the method of “tell-show-do” (R = −0.273; *p* ≤ 0.001). In addition, age correlates with the materials used in working with children. For example, the use of glass-ionomer cement negatively correlates with the age of the subjects (R = −0.171; *p* = 0.002) but age positively correlates with composites (R = 0.158; *p* = 0.005). Unlike those employed in the private sector, dentists employed in a health center more often used glass-ionomer cement in children (R = 0.148, *p* = 0.008). The weekly frequency of children in dental offices was positively correlated with the contract with the Croatian Health Insurance Fund (R = 0.237; *p* ≤ 0.001). The final score obtained in the questionnaire on the attitude of dentists toward working with children correlates with the female gender of the respondents (R = −0.103; *p* = 0.035). Furthermore, the final score was positively correlated with the weekly frequency of children in the dental practice (R = 0.206; *p* ≤ 0.001), the opinion that they gained enough experience to work with children during university (R = 0.355; *p* ≤ 0.001), the acquisition of knowledge to work with children by attending professional seminars (R = 0.197; *p* = 0.027), using the “tell-show-do” method (R = 0.122; *p* = 0.030), and also with performing endodontic treatment of deciduous teeth (R = 0.172; *p* = 0.002).

## 4. Discussion

Of a total number of 361 doctors who participated in this study, only a small percentage of dentists (12.43%) have a positive attitude toward working with children. In addition, only a small percentage of respondents attend additional education on working with children after their college education. As in most countries in the world, there is a shortage of pediatric dentists in Croatia [13], so it is extremely important that general dentists have a positive attitude toward working with children [14]. In this study, as many as 74.51% of the respondents were women, which is in line with the total number of female dentists in Croatia. In the register of dentists in Croatia, 5066 dentists are registered [13], of which 66.4% are women, indicating that dentistry in Croatia is predominantly a female occupation [13]. With the exception of Croatia, the upward trend of female dentists is visible in the majority of European countries in which dentistry has, until recently, been a predominantly male profession [15,16]. In the US, dentistry is still a predominantly male profession but there is a consistent increase in the number of women in the profession. In 2010, 24.5% of all dentists in the United States were women; in 2016 that number rose to 29.8% [17].

Research conducted in India has shown that most patients feel more relaxed when treated by a female dentist [18]. Due to their psychological profile, females establish a closer relationship with the patient and show more understanding and compassion [16,19,20]. Women are by nature considered more caring, approachable, and expressive compared to men [18]. Furthermore, women spend more time getting to know and communicating with the patient and pay more attention to explaining the treatment and diagnosis [18]. This study noticed an association between the female gender and the attitude toward working with children. In addition, research in Israel has shown that more females choose to specialize in pediatric dentistry than males. In this study, the tell-show-do method is the most common behavior guidance technique for pediatric dental patients, which is in accordance with other studies conducted worldwide [21]. Additionally, female dentists are more likely to use the tell-show-do method than men [19]. In this study, the use of the tell-show-do method negatively correlated with the age of the respondents, thus showing more patience in younger dentists when working with children.

A study conducted by Riley and co-workers found that women dentists devoted more time to preventive measures and procedures in treating children and adult patients [22]. In this study, the attitude toward working with children was associated with the female gender, which is in agreement with other studies [18,19,22]. In addition, the positive attitude of a dentist toward working with children also depends on the level of education of the dentist (Figure 2). Similar results are shown by a 2010 study conducted by Garg et al., in New York, which states that additional training and access to counseling increase the willingness of dentists to work with children [10]. More than half of the respondents in this research, (57.06%) believe that they did not gain enough experience to work with children during their university education; however, the perception that they did not gain enough experience during their university education does not correlate with further education in pediatric dentistry in professional seminars (R = 0.133; *p* = 0.073). In the study mentioned above by Garg et al., most dentists surveyed received additional education in lectures and professional seminars; in contrast, in our study, only 8% of surveyed dentists stated that they improved their knowledge in professional workshops [10].

The fact that 17% of respondents stated that they only improve their work with children by working in practice—and that attitude is negatively correlated with going to professional seminars (R = −0.440; *p* < 0.001) and searching for additional literature on the Internet (R = −0.383; *p* < 0.001)—further supports the lack of interest seen among some dentists on this topic.

Restorative treatments (82.72%) are the most common therapeutic procedures in children, followed by extractions, and endodontic treatments. These procedures are performed to prevent the progression of caries and pulp involvement and, finally, the loss of the deciduous teeth, which could affect further growth and development of the jaw and stomatognathic system [23]. A study conducted by Duangthip et al., in 2017 states that restorative procedures in children have a high success rate (42% for composites and amalgam fillings and 33% for conventional glass-ionomer cement) [23].

In this study, the most common frequency of children in dental offices is 5 to 10 weekly (40.10%). The weekly frequency of children in dental offices is positively correlated with the contract with the Croatian Health Insurance Fund (R = 0.237; *p* ≤ 0.001). It could be explained by the fact that children in the Republic of Croatia enjoy complete health care, and the Croatian Health Insurance Fund covers all dental treatment costs [24]. Despite this, Croatia is a country with a high dmft/DMFT index [25].

In this study, the most represented children’s age group in dental offices was from 5 to 10 years (64.54%), and the least is from 0 to 5 years (3.32%). The American Dental Association (ADA) recommends that the first child checkup in the dental office should be done within six months of tooth eruption, no later than the child’s first birthday [26]. Contrary to that, in the study on pregnant women by Gavic et al., in Croatia, the most significant number of mothers (53.23%) mistakenly believe that the right time to take a child to the dentist is when all deciduous teeth erupt [27]. Therefore, the low representation of the youngest age group of children in dental offices in Croatia is not surprising.

In this study, dentists mostly answered that the therapy of young permanent teeth is the most challenging aspect of their work with children. The term young permanent teeth is used for permanent teeth erupted in the oral cavity in which the growth and development of the root are not completed. Therefore, it is of great importance to preserve the vitality of the pulp until root development is complete (Table 1). Young permanent teeth with necrotic pulp and periapical processes are a significant treatment challenge due to the lack of an apical barrier and the impact on periodontal tissues [28,29].

Of the materials used for fillings, glass-ionomer cement was primarily used (57.62%), and somewhat less often were the composites (37.12%) (Table 1). The usage of glass-ionomer cement negatively correlates with the age of the subjects (R = −0.171; *p* = 0.002), which means that younger dentists more often use glass ionomers. This could be explained by the fact that glass-ionomer cement is a slightly newer material, so younger dentists are better acquainted with its benefits [30].

When we look at the questions in the questionnaire about the attitude towards working with children, dentists least agreed with the statements that the financial compensation for the treatment of children is adequate (Figure 1). It is interesting that the answers to this question are slightly negatively correlated with dentists who own private practices (R = −0.139, *p* = 0.004). As mentioned earlier, the fund provides treatment for children free of charge, which private dentists do not have access to. In addition, they did not agree with the statement that the stress when treating children is no different from stress when treating adult patients (Figure 1). This is in the accordance with the results of previous studies, where therapists were highly stressed while performing pedodontic treatment [31,32].

This study has certain limitations. Namely, this is a cross-sectional study that cannot establish a cause-and-effect relationship or analyze behavior over time. In addition, the difference in the attitudes toward the treatment of adults and children should be examined. Perhaps some dentists have no more patience with treating any age group due to overwork.

To the best of our knowledge, this study is the first in the Republic of Croatia about the attitudes of dentists toward treating children. In addition, based on the results of this study, more professional continuing education courses on treating children could be set. Namely, dentists need to approach children as patients in a particular way, be patient, and thus facilitate the procedures for children, which is gained through continuous improvement of knowledge and experience.

## 5. Conclusions

The availability of oral health care provided by professionals is an essential children’s right. Unfortunately, not all children have access to a pediatric dentist’s oral healthcare, so the willingness of general dentists to work with children is crucial. Furthermore, the positive attitude of general dentists toward treating children depends on knowledge; therefore, continuous improvement of knowledge and experience is essential. Therefore, general dentists should develop skills to enhance their ability to work with children.

## Figures and Tables

**Table 1:** Survey of respondents considering children in their dental office.

	Answers	N (%)
Frequency of children in dental offices	0–5 children/week	93 (25.76%)
	5–10 children/week	148 (40.10%)
	10–15 children/week	66 (18.28%)
	>15 children/week	54 (14.96%)
The most frequent age group of children in the dental office	0–5 years	12 (3.32%).
	5–10 years	233 (64.54%)
	10–15 years	63 (17.45%)
	˃15 years	53 (14.69%)
Office’s equipment to make them more accessible and comfortable for children	A special corner for children in the waiting room	56 (15.51%)
	Children’s dental chair	2 (0.55%)
	Toys and children’s multimedia content	108 (29.92%)
	Nothing	195 (54.02%)
Representation of procedures on children in dental offices	Oral examination	31 (9.57%)
	Restorative interventions	268 (82.72%)
	Extractions	6 (1.85%)
	Endodontic treatment	13 (4.01%)
	Other	6 (1.85%)
The most commonly used material in restorative procedures in children	Glass-ionomer cement	208 (57.62%)
	Dental composite resins	134 (37.12%)
	Compomer	18 (4.99%)
	Dental amalgam	1 (0.28%)
The most difficult therapeutic procedures in children	Administration of local anesthesia	47 (14.64%)
	Cavity preparation	35 (10.90%)
	Work field insulation	193 (60.12%)
	Extraction	14 (4.36%)
	Pulpotomy	32 (9.98%)
Child behavior to be the most challenging for work	Rebellious	201 (55.68%)
	Pretentious	39 (10.80%)
	Scared	106 (29.36%)
	Restrained	15 (4.16%)
The most difficult aspects of working with children	Behavior management of children	136 (42.63%)
	Physical effort	59 (18.50%)
	Parental behavior	111 (34.80%)
	Financial cost-effectiveness	13 (4.79%)
Reasons for referring a child to the pediatric dentist specialist	Theoretical and practical inadequacies in education	274 (86.44%)
	Difficulties in diagnosis	10 (3.15%)
	Child non-cooperation	16 (5.05%)
	Age less than 3 years	7 (2.21%)
	Difficulties in restorative procedures	10 (3.15%)

## Data Availability

The data that support the findings of this study are available from the corresponding author upon reasonable request.
